# Charge Properties and Electric Field Energy Density of Functional Group-Modified Nanoparticle Interacting with a Flat Substrate

**DOI:** 10.3390/mi11121038

**Published:** 2020-11-26

**Authors:** Luyu Deng, Liuyong Shi, Teng Zhou, Xianman Zhang, Sang W. Joo

**Affiliations:** 1 Mechanical and Electrical Engineering College, Hainan University, Haikou 570228, China; dengluyulvqj@foxmail.com (L.D.); shiliuyong@hainu.edu.cn (L.S.); xianman213@163.com (X.Z.); 2 School of Mechanical Engineering, Yeungnam University, Gyeongsan 38541, Korea

**Keywords:** nanoparticle, charge density, electric field energy density, interaction

## Abstract

Functionalized nanofluidics devices have recently emerged as a powerful platform for applications of energy conversion. Inspired by biological cells, we theoretically studied the effect of the interaction between the nanoparticle and the plate which formed the brush layer modified by functional zwitterionic polyelectrolyte (PE) on the bulk charge density of the nanoparticle brush layer, and the charge/discharge effect when the distance between the particle and the plate was changed. In this paper, The Poisson–Nernst–Planck equation system is used to build the theoretical model to study the interaction between the nanoparticle and the plate modified by the PE brush layer, considering brush layer charge regulation in the presence of multiple ionic species. The results show that the bulk charge density of the brush layer decreases with the decrease of the distance between the nanoparticle and the flat substrate when the interaction occurs between the nanoparticle and the plate. When the distance between the particle and the plate is about 2 nm, the charge density of the brush layer at the bottom of the particle is about 69% of that at the top, and the electric field energy density reaches the maximum value when the concentration of the background salt solution is 10 mm.

## 1. Introduction

Surface modification technology refers to the modification of nanoparticles or devices, and the brush layer is formed on the surface of the object by zwitterionic functional groups, which is generally called a functional polyelectrolyte (PE) brush layer. This technology has been widely used in ion rectification [1,2,3,4,5], energy collection [6,7,8], material transport [9,10,11,12], DNA sequencing [13,14], etc. When the nanoparticle (or plate) modified by the PE brush layer is placed in salt solution, changing the pH value in the solution domain or the concentration of the background salt solution will change the charge properties in the PE brush layer, resulting in some unique and special phenomena; for example, increasing the through-hole rate of nanoparticles through nanopores [11,15,16], changing the ion selectivity and ion transport efficiency of nanochannels [1,17,18,19]. The fundamental understanding of the physics behind these special phenomena is essential for the development of functionalized nanofluidics. Despite the growing experimental and theoretical efforts, the understanding of various phenomena of a functional PE brush layer in salt solution remains limited. For example, whether the charge characteristics of a nanoparticle modified by a PE brush layer will affect the absorption of the nanoparticle by cells and how the electrostatic interaction between the nanoparticle modified by PE brush layer and plate in salt solution will affect the brush layer properties of nanoparticle.

When the PE brush-modified nanoparticle comes into contact with the electrolyte solution, the volume charge is generated in the brush layer due to the ion enrichment and the protonation/deprotonation reaction of functional groups inside the brush layer. The change of volume charge density in the PE brush layer is mainly controlled by local concentration of H^+^. Adjusting the concentration and pH of the background salt solution is not the direct cause of the change of the volume charge density, but by affecting the change of the local concentration of H^+^ ion in the brush layer to achieve the purpose of controlling the volume charge density, and finally affecting the electric field energy change in the PE brush layer. For example, silica nanoparticles of different sizes are placed in salt solution to change the size of the surface charge density of the particles by adjusting the local concentration of H^+^ in contact with the particle surface by changing the pH, background salt solution concentration and other conditions [20,21]. Alternatively, the brush charge density of the channel wall can be changed by adjusting the thickness of the brush layer on the wall to change the local concentration of H^+^ contacted by the brush layer [1,22,23]. When two charged objects are close to each other, there will be interaction between them. Therefore, the local solution properties such as ionic concentrations in the gap between the two interacting objects are different from those when the two objects are sufficiently away from each other; for example, when the distance between two negatively charged objects is less than 10 nm, and the cations in their gap are enriched by interaction. Spatially inhomogeneous ion concentration is generated around the interacting object, which results in uneven distribution of space charge density around the object [24,25,26,27,28,29,30]. However, most studies are still based on the interaction system of silica and metal materials, or assume that the surface charge properties of two interacting objects remain at their volume values. In the practical application of the functionalized nanofluidics, when nanoparticle modified by a PE brush interact with nanochannel walls (or nanopore), the local ion concentration in the PE brush may be significantly different from that in other locations.

The objective of this study is to discuss the effect of different spacing, pH value, and background salt solution concentration on the charge density and electric field energy density of the brush body at the bottom of the particle when the PE brush-modified nanoparticles interact with the plate. The functional groups in the PE brush layer react with contact H^+^ and generate positive/negative ion groups in the brush layer, which changes the bulk charge density and electric field energy density in the brush layer. By changing the above conditions to enhance or weaken the interaction between the particle and the plate, the local H^+^ ion concentration in the PE brush layer at the bottom of the particle changes, which affects the chemical reaction equilibrium in the brush layer, and changes the number of positive and negative ion groups. Because the nanoparticles modified by the PE brush layer have been widely used in colloid science, biomolecule transport and drug delivery [21,31,32,33,34,35,36,37], and many nanofluid devices (such as nanopores and nanochannels) use PE brush layer as wall coating [38,39,40], we investigate the charge characteristics of nanoparticles modified by a PE brush layer interacting with a flat plate. In contrast to most previous studies on surface charge density changes of silica particle, we analyzed the effects of local pH and background salt concentration on bulk charge density using a multi-ion charge regulation model, as well as the changes of electric field energy density in brushed nanoparticle brushes at different distances to study the charge/discharge effect between particles and plate [41,42,43].

## 2. Model Description 

**Figure 1:**
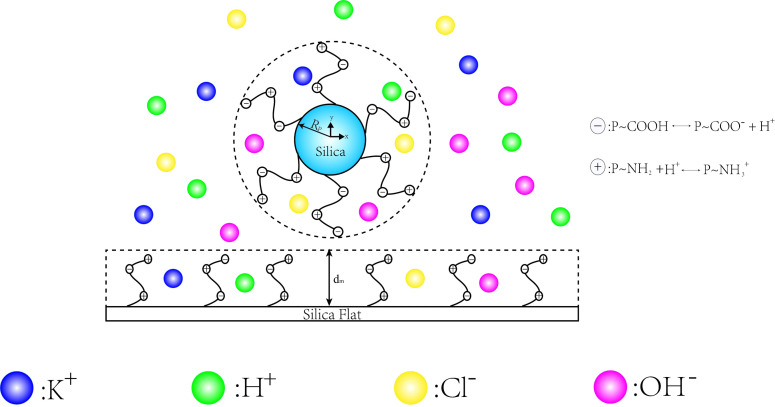
The schematic diagram of interaction between brush nanoparticle and brush planar substrate in the solution domain containing K^+^, H^+^, Cl^−^, OH^−^ ionic species.

As schematically shown in Figure 1, we consider a R_P_ nanoparticle of radius suspended on a flat substrate in a solution domain containing N ions. The functional groups P–NH_2_ and P–COOH were used to modify the surface of nanoparticles and plates to form PE brushes with a thickness of dm. The distance between the bottom end of the nanoparticle and the plate is w. This study selected KCl solution with concentration of C_KCl_ as background salt solution in the electrolyte, KOH and HCl are used to adjust the pH value of the electrolyte solution. Therefore, there are four kinds of ion in the solution: K^+^, H^+^, Cl^−^, OH^−^.

**Figure A1:**
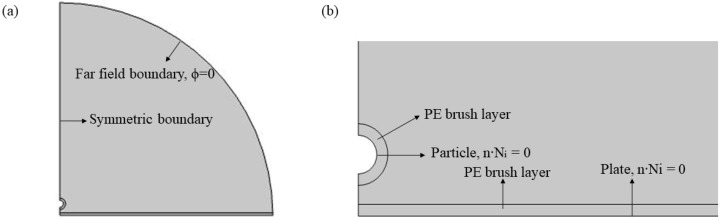
The structure diagram of the model in software, (**a**) structural integrity, (**b**) local structure.

In this study, a two-dimensional axisymmetric model was used to set the center of the rectangular coordinate system at the center of the nanoparticle. Due to the existence of many ions in the solution domain, the electrostatic and ion mass transport are controlled by the steady state Poisson–Nernst–Planck (PNP) equation,

(1) $$-\varepsilon_{0}\varepsilon_{f}\nabla^{2}\phi =\rho_{e}=F\sum_{i=1}^{4}z_{i}c_{i}$$

and,

(2) $$\nabla \cdot N_{i}=\nabla \cdot \left(-D_{i}\nabla c_{i}-z_{i}\frac{D_{i}}{RT}Fc_{i}\nabla \phi \right)=0,\;i=(1,2,3,4)$$

where, *c_i_*, z*_i_* and *D_i_* represent the molar concentration, valence and diffusion coefficient of the *i*th ionic species (*i* = 1 for H^+^, *i* = 2 for K^+^, *i* = 3 for Cl^−^ and *i* = 4 for OH^−^), *ε*_0_ is the permittivity of vacuum, *ε*_f_ is the relative dielectric constant of the electrolyte solution, *T* is the absolute temperature of electrolyte solution, *ϕ* is the potential inside electrolyte solution, *F* is the Faraday constant, *ρ*_e_ is the space charge density inside the electrolyte solution, *R* is universal gas constant. On the rigid surfaces of the nanoparticle, normal ionic flux for each species is zero, *n*∙N*_i_*= 0 (*i* = 1, 2, 3, 4). The electric potential *ϕ* at infinity is set to 0. In the brush layer of the nanoparticles, bulk charge density of the brush layer boundary condition, −*ε*_0_*ε*_f_∙*n*∙∇*ϕ* = *ρ*_m_, is imposed, as shown in Figure A1.

Most organic molecular chains in biological systems usually have pH-regulating properties, which indicate that their bulk charge density depends on the local concentration of H^+^ changes. Therefore, when the pH of the solution changes, the PE brush layer with zwitterionic functional groups can carry out the following protonation/deprotonation reactions,

(3) $$P–\mathrm{COOH}↔P–{\mathrm{COO}}^{-}{+H}^{+}$$

(4) $$P–{\mathrm{NH}}_{2}{+H}^{+}↔P–{\mathrm{NH}}_{3}{}^{+}$$

Assume that the equilibrium constants of the two reactions are K_a_ and K_b_, respectively,

(5) $$K_{a}=\frac{\left[P–{\mathrm{COO}}^{-}\right]\left[H^{+}\right]}{\left[P–\mathrm{COOH}\right]}{\;\mathrm{and}\;K}_{b}=\frac{\left[P–{\mathrm{NH}}_{3}{}^{+}\right]}{\left[P–{\mathrm{NH}}_{2}\right]\left[H^{+}\right]}$$

where, [H^+^] denotes local the concentration of H^+^ in the brush layer, [H^+^] = 10^−pH^exp(−*ϕ*/*ϕ*_0_), *ϕ*_0_ = RT/F. [P–COO^−^], [P–COOH], [P–NH_2_], [P–NH_3_^+^] denotes the bulk density of each electrolyte group in the brush layer respectively. It can be shown that the volume charge density of the brush layer can be expressed as,

(6) $$\begin{array}{l} \rho_{m}=1000F\left(\left[P–{\mathrm{NH}}_{3}{}^{+}\right]-\left[P–{\mathrm{COO}}^{-}\right]\right)\\ \;=1000F\left(-\frac{K_{a}\Gamma_{a}}{K_{a}+\left[H^{+}\right]}+\frac{K_{b}\Gamma_{b}\left[H^{+}\right]}{{1+K}_{b}\left[H^{+}\right]}\right) \end{array}$$

The net volume densities of acid electrolyte group and alkaline electrolyte group are *Γ*_a_ = [P–COO^−^] + [P–COOH] = Nσ_m_/1000 d_m_n_a_, *Γ*_b_ = [P–NH_3_^+^] + [P–NH_2_] = Nσ_m_/1000 d_m_n_a_, respectively. σ_m_ is the grafting density of the brush layer on the particle surface (the number of electrolyte group chains connected on the particle surface in unit volume, generally from 0.05 to 0.6 chains/nm^2^) [5,44,45], n_a_ is the Avogadro constant, N is the number of electrolyte groups on a single chain in the brush layer. To solve the coupled Equations (1) and (2), appropriate boundary conditions are required. Along the y axis, axial symmetry boundary conditions for electric potential and for each ionic concentration are applied. Far away from the charged nanoparticle and plate, we assume that the ionic concentration of each species maintains its bulk concentration, c*_i_* = C*_i_*_0_, *i* = 1, 2, 3, 4. According to the electroneutral condition, the volume concentration of various ions in the solution is as follows [11],

(7) $$C_{10}=10^{-\mathrm{pH}+3}\;{\mathrm{and}}^{}\;C_{40}=10^{-\left(14-\mathrm{pH}\right)+3}$$

(8) $$C_{20}=C_{\mathrm{KCl}}\;{\mathrm{and}}^{}\;C_{30}=C_{\mathrm{KCl}}+C_{10}-C_{40}\;{\mathrm{when}}^{}\;\mathrm{pH}\leq 7$$

(9) $$C_{20}=C_{\mathrm{KCl}}-C_{10}+C_{40}\;{\mathrm{and}}^{}\;C_{30}=C_{\mathrm{KCl}}\;{\mathrm{when}}^{}\;\mathrm{pH}>7$$

Since the bulk charge density of PE brush layer is affected by local concentration of H^+^, we define the average bulk charge density *ρ_n_* of PE brush layer as follows,

(10) $$\rho_{n}=\frac{1}{d_{m}}\int_{R_{P}}^{R_{P}+d_{m}}\rho_{m}dy$$

Therefore, the total charge density ρ in the PE brush layer is the sum of the charge density *ρ*_e_ generated by the local ion concentration in the brush layer and the average bulk charge density *ρ_n_* of the PE brush layer,

(11) $$\rho =\rho_{e}+\rho_{n}$$

The relationship between the electric field energy density ω and the total charge density *E* can be obtained by the formula of electric field energy density in the electrostatic field,

(12) $$\omega =\frac{1}{2}\varepsilon_{0}E^{2}$$

This manuscript discusses the change of electric field energy density in PE brush layer when the particle is close to the plate. The relationship between electric field intensity *E* and total charge density *ρ* in PE brush layer can be obtained by the differential form of the Gauss theorem,

(13) $$\nabla \cdot E=\frac{\rho }{\varepsilon_{0}}$$

integral on both sides of Equation (13),

(14) $$\int_{V}\nabla \cdot \vec{E}dV=\int_{V}\frac{\rho }{\varepsilon_{0}}dV$$

(15) $$\int_{V}\nabla \cdot \vec{E}dV=\oint_{S}\vec{E}dS$$

(16) $$\oint_{S}\vec{E}dS=\frac{1}{\varepsilon_{0}}\int_{V}\rho dV$$

From the above derivation, the change of the energy density of the electric field *E* in the PE brush layer can be described by discussing the total charge density *ρ*.

Debye length *λ*_D_ of electric double layer (EDL) on particle surface [46],

(17) $$\lambda_{D}=\sqrt{\varepsilon_{0}\varepsilon_{f}RT/\sum_{i=1}^{4}F^{2}z_{i}{}^{2}C_{i0}}$$

In order to avoid the influence of the boundary of the solution domain on the results, we install the far-field boundary of the solution domain so that it is much larger than the particle radius R_P_ (about 50 times of R_P_ + *λ*_D_). Free triangular mesh within the model is adopted, the mesh vertex is 37,177, and the number of triangles is 72,445. 

## 3. Results and Discussion

The two-dimensional axisymmetric model described above is implemented numerically by COMSOL Multiphysics (version 4.0, COMSOL Inc., Stockholm, Sweden). Through the numerical calculation, we explored the interaction between the nano particle modified by the PE brush layer and the plate under different pH and background salt concentrations. Meanwhile, we discussed the changes of volume charge density and electric field energy density in the PE brush layer. Physical parameters employed in the simulations were *R* = 8.31 J/(mol·K), ε_0_ = 8.854 × 10^−12^ CV^−1^m^−1^, *ε*_f_ = 80, *F* = 96,490 C/mol, *T* = 298 K, respectively. The relative permittivity ε_f_ and the diffusivity of the ionic species i, D_i_, inside the Nanoparticle brush layers were the same as those outside them. The diffusion coefficients of H^+^, K^+^, OH^−^, Cl^−^ are D*_i_* (*i* = 1, 2, 3, 4) = 9.31 × 10^−9^, 1.96 × 10^−9^, 5.30 × 10^−9^, 2.03 × 10^−9^, respectively. The nanoparticle brush layer was uniformly structured and the deformation of that layer was neglected, which was valid if the repeated unit of biomimetic polyelectrolyte groups, N, was not too high (e.g., N ≤20) [11]. Grafting density of the biomimetic electrolyte in the brush layer was σ_m_ = 0.15 chains/nm^2^, number of electrolyte groups on single-chain N = 20, p*K*_a_ = −log *K*_a_ = 2.2 (α-carboxyl), p*K*_b_ = −log *K*_b_ = −8.8 (α-amino) [47].

**Figure 2:**
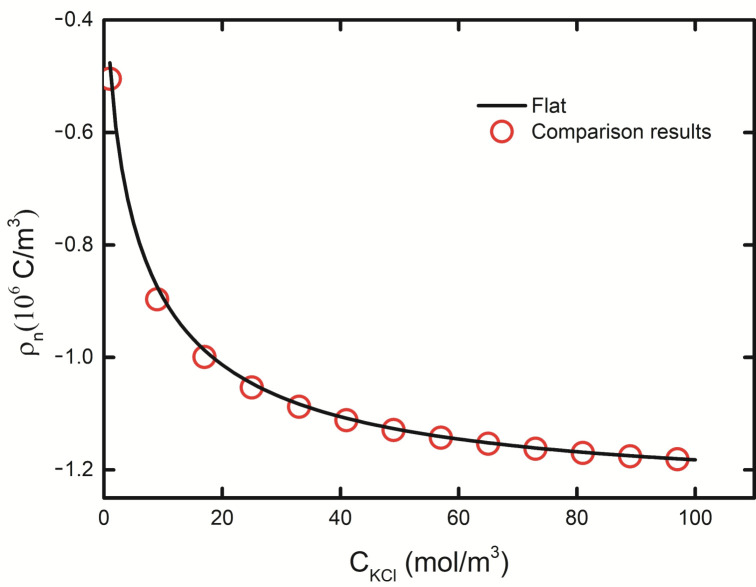
The fluctuating curve of brush layer charge density depending on background salt solution concentration of the plate, shown as the black solid line, and the red dot represents the analysis result in [1], pH = 7.

In order to verify the correctness of the designed model, the curve of the brush layer charge density of the channel wall with the background salt solution was obtained in [1] when the gate voltage was 0. For the convenience of verification, we calculated the curve of the volume charge density of PE brush layer of the plate with the concentration of background salt solution when pH = 7 in the solution domain, and compared it with the results in [1]. It is obvious that our simulated structure (black solid line) completely coincides with the analysis result (red dot line) in [1], as shown in Figure 2. The accuracy and reliability of the model are verified.

### 3.1. Interaction between Particle and Plate: pH Effect

**Figure 3:**
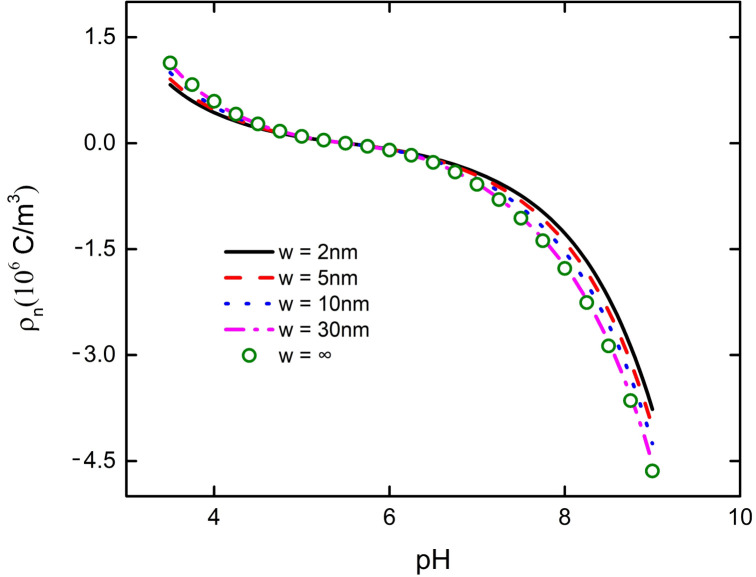
The change curve of bulk charge density of the polyelectrolyte (PE) brush layer at the bottom of the nanoparticle with pH under different spacing between particle and plate at C_KCl_ = 1 mM.

The influence of interaction between nanoparticle and plate on the bulk charge density of PE brush layer is solved by numerical calculation. Figure 3 describes when the concentration of the background salt solution is fixed at 1 mM, the volume charge density of the PE brush layer at the bottom of the nanoparticle varies with the solution pH at different distances between the particle and the plate. When the particle and the plate are close to each other, there will be interaction, which will affect the decrease of the brush layer charge density near the plate, as shown in Figure 3. With the increase of the distance between the particle and the plate, the interaction effect is gradually weakened. When the distance between the particle and the plate w ≥ 30 nm, the interaction between the particle and the plate does not occur (pink dot and green dot basically overlapped in Figure 3). From Figure 3, it can be judged that at the beginning, the bulk charge density in the PE brush layer is positive, and it decreases with the increase of pH. When the pH increases to 5.5, the value of the volume charge density decreases to zero. As the pH continues to increase, the polarity reversal of the bulk charge density in the PE brush layer becomes negative, and it increases with the increase of pH. The pH value at which the polarity reversal occurs is generally referred to as the zero-potential point (IEP). Because of the existence of the IEP point, when pH = 5.5 in the solution, the bulk charge density of the PE brush layer between particle and plate is 0. Therefore, there is no interaction between the particle and plate. This explains why the influence of the channel wall on the particle transport efficiency is weakened when the PE brush layer modified nanochannel is at the IEP point.

**Figure 4:**
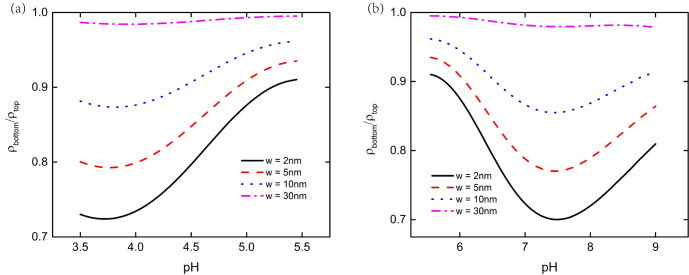
The ratio of bulk charge density of PE brush layer at the bottom and the top of particle as a function of pH under different distances between the particle and the plate at C_KCl_ = 1 mM. (**a**) At pH < 5.5, the charge of PE brush layer is positive (**b**) at pH > 5.5, the charge of PE brush layer is negative.

**Figure 5:**
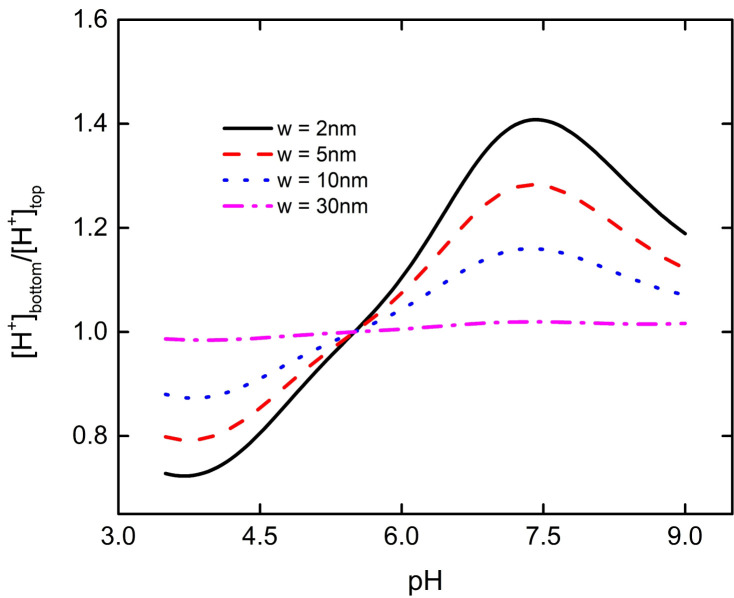
The ratio of the local concentration of H^+^ at the bottom and the top of the particle as a function of pH under different distances between the particle and the plate at C_KCl_ = 1 mM.

We use the ratio of bulk charge density of the PE brush layer at the top and bottom of the nanoparticle to show the influence of interaction between nanoparticles and the flat plate more clearly, as shown in Figure 4. Figure 4a depicts when pH < 5.5, the charge density ratio of the PE brush layer at the bottom of the particle to the top of the particle first decreases and then increases, reaching the lowest value at pH = 3.7. Figure 4b depicts that when pH > 5.5, the charge density ratio of the PE brush layer at the bottom and top of the particle also decreased first and then increased, reaching the lowest value at pH = 7.4. When there is no interaction between nanoparticle and plate, the charge density ratio of the PE brush layer at the top and bottom of the particle is 1. When the interaction between the particle and the plate is very weak, which occurs when the separation distance is much larger than the brush layer thickness, the influence of the flat plate on the bulk charge density of the PE brush layer at the top of the particle is ignored. However, the interaction between the bottom of the particle and the plate is very obvious, especially when the distance is much smaller between the particle and the plate. For example, when w = 2 nm, at the lowest point of the particle in Figure 4a, the bulk charge density of the PE brush layer at the bottom of the particle is about 72% of that at the top. At the lowest point of the particle in Figure 4b, the bulk charge density of the PE brush layer at the bottom of the particle is about 69% of that at the top. It can be seen from Equations (3) and (4) that the bulk charge density of the PE brush layer of the nanoparticle is affected by local concentration of H^+^. The change of local concentration of H^+^ will lead to the change of P–COO^−^ and P–NH_3_^+^ ion groups on the particle’s surface, which will lead to the change of bulk charge density of the PE brush layer. Based on the above conclusion, we draw the ratio curve of the local concentration of H^+^ the bottom end of the particle to the top of the PE brush layer, as shown in Figure 5. From Figure 5, it can be seen that when pH < 5.5, the PE brush layer is positively charged, and the charge density of the brush layer decreases with the decrease of concentration of H^+^. At pH = 3.7, the ratio of the local concentration of H^+^ the bottom end of the particle to the top of the particle reaches the minimum value, resulting in the minimum charge density ratio. When pH > 5.5, the PE brush layer is negatively charged, and the charge density of the brush layer increases with the decrease of concentration of H^+^. At pH = 7.4, the ratio of the local concentration of H^+^ from the bottom end of the particle to the top of the particle reaches the maximum, resulting in the minimum charge density ratio.

**Figure 6:**
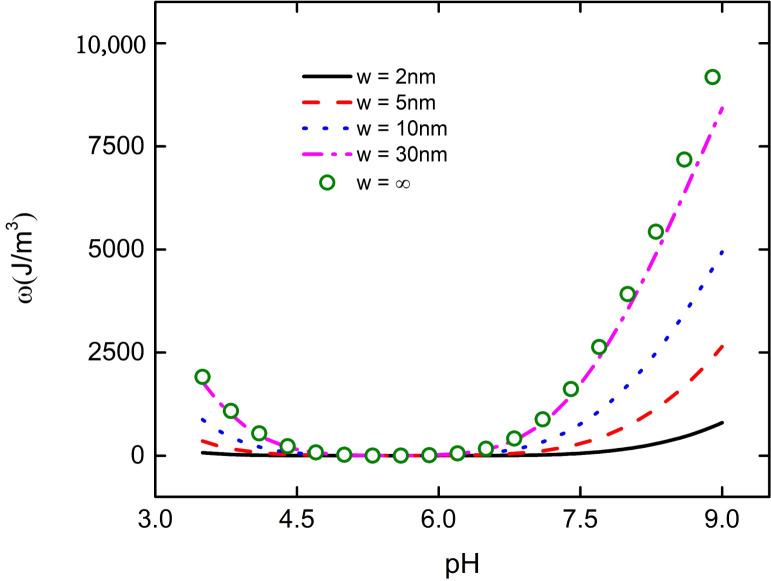
The curves of electric field energy density with pH in the PE brush layer at the bottom of nanoparticle with different distances between particle and plate at C_KCl_ = 1 mM.

**Figure 7:**
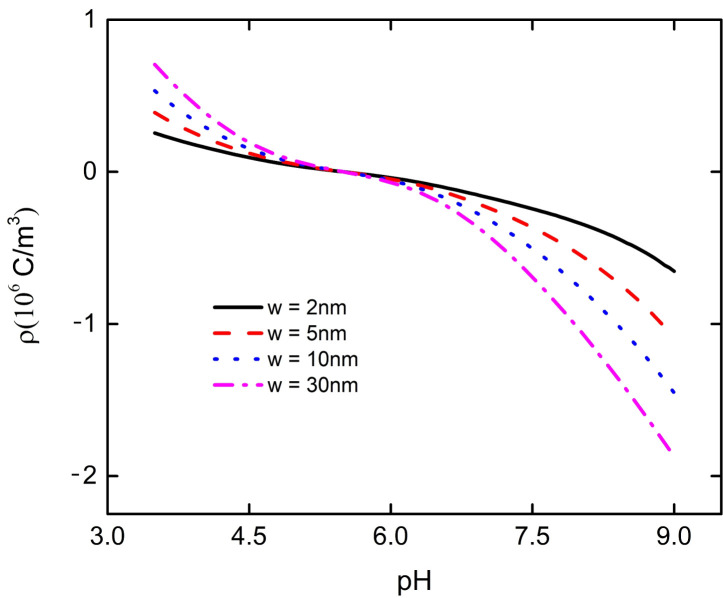
The change curve of total charge density with pH in the PE brush layer at the bottom of nanoparticle with different distances between particle and plate at C_KCl_ = 1 mM.

The interaction between nanoparticles and flat plate will not only affect the bulk charge density of the PE brush layer at the bottom of the particle, but also change the electric field energy density. Under the fixed distance between particle and plate, the electric field energy density at the bottom of the particle decreases with the increase of pH. When the pH increases to 5.5, the electric field energy density at the bottom of the particle decreases to 0, and then increases with the increase of pH, as shown in Figure 6. Since the total charge density ρ at the bottom of the particle decreases with the increase of pH, when the pH increases to 5.5, the charge at the bottom of the particle changes from positive to negative, and then increases with the increase of pH, as shown in Figure 7. From Equations (12) and (16), it can be seen that the change of total charge density ρ leads to the change of electric field intensity E, which affects the change of electric field energy density in the PE brush layer at the bottom of the particle. From Figure 6, it can be seen that the smaller the distance between the particles and the plate, the smaller the electric field energy density at the bottom of the particles. For example, when w = 2 nm, the electric field energy density at the bottom of the particle is only 1500 J/ m^3^ at pH = 9; when w = ∞, it is close to 10,000 J/ m^3^ at pH = 9. Due to the interaction between the nanoparticle and the flat plate, the ability of the PE brush layer at the bottom of the particle to attract various ions is weakened, resulting in the decrease of the total charge density and the electric field energy density at the bottom of the particle. This explains why the velocity of macromolecular organic compounds passing through nanopores varies with the pH of the solution.

### 3.2. Interaction between Particle and Plate: Background Salt Solution Concentration

**Figure 8:**
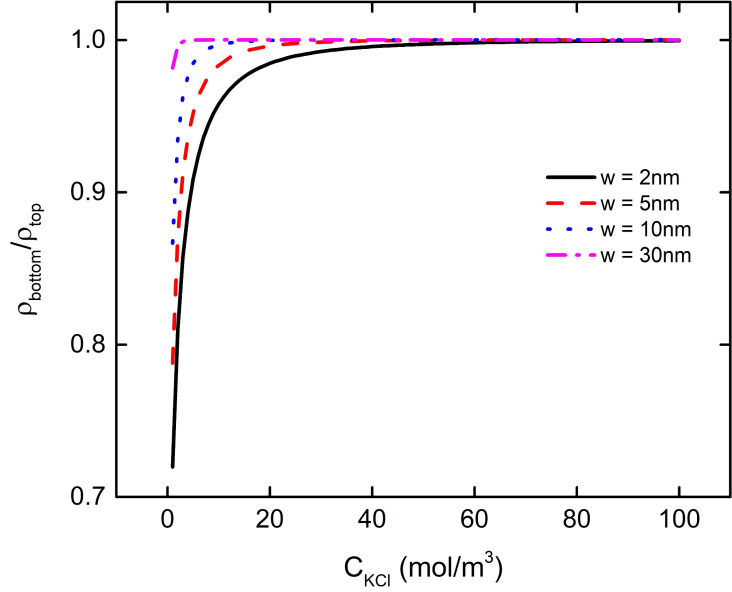
The ratio of bulk charge density of the PE brush layer at the bottom and top of the particle as a function of C_KCl_ under different distances between particle and plate at pH = 7.

**Figure 9:**
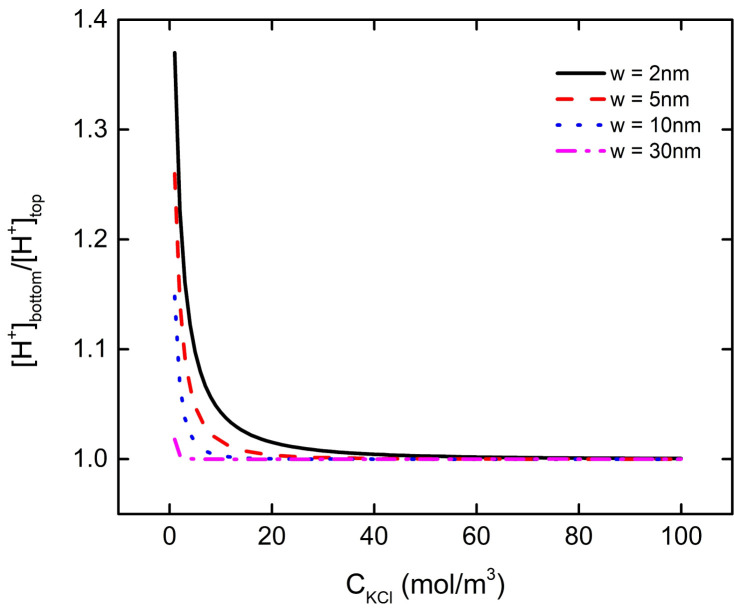
The ratio of the local concentration of H^+^ at the bottom and the top of the particle as a function of C_KCl_ under different distances between particle and plate at pH = 7.

The results of the previous discussion show that the interaction between nanoparticle and plate will affect the bulk charge density and electric field energy density of the particle PE brush layer. Moreover, the degree of their interaction is affected by the pH of the solution as well as the distance between the particles and the plate. In this section, we fasten pH = 7 in the solution to discuss the influence of the concentration of background salt solution on the interaction between nanoparticle and plate. With the increase of the concentration of the background salt solution, the ratio of bulk charge density of the PE brush layer at the bottom and top of the particle increases continuously, as shown in Figure 8. When the concentration of the background salt reaches a certain value, the ratio is close to 1. For example, when w = 2 nm, 5 nm, 10 nm, 30 nm, the ratio of charge density at the bottom and the top of the brush layer reaches about 1 at C_KCl_ = 50 mM, 30 mM, 13 mM, 3 mM. Since there is a negative charge of the brush layer between the nanoparticle and the plate, when the concentration of the background salt solution is low, the local concentration of H^+^ in the PE brush layer at the bottom of the particle is larger than that at the top of the particle under the interaction between the particles and the plate, which makes the P–COO^−^ group in the brush layer at the bottom of the particle less than that at the top of the particle. With the increase of the concentration of background salt solution, the concentration of K^+^ in the solution increases gradually, which leads to the increase of local concentration of K^+^ between the bottom of the nanoparticle and the plate, and weakens the influence of interaction between the particle and plate on the local concentration of H^+^. When the concentration of background salt solution reaches a certain level, the interaction between particle and plate no longer affects the local H^+^ concentration at the bottom of the particle. At this time, the local H^+^ concentration ratio between the bottom and top of the particles is close to 1, which does not change with the increase of background salt solution concentration, as shown in Figure 9.

**Figure 10:**
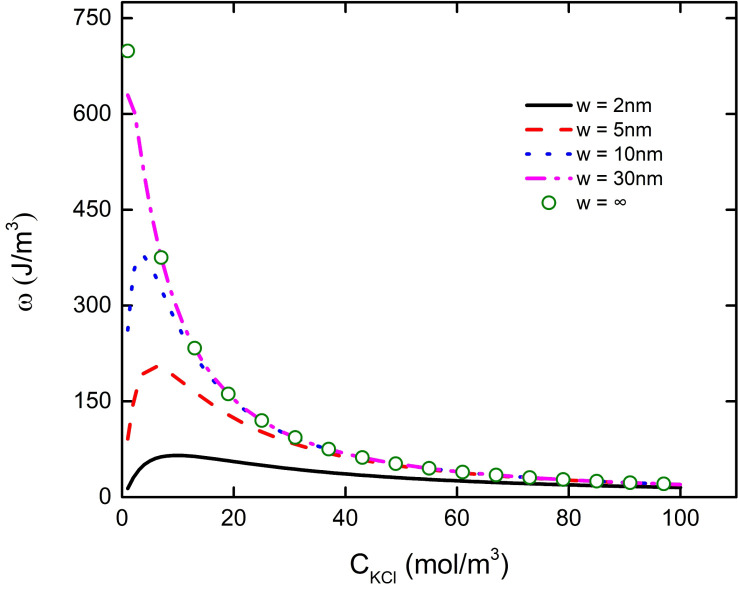
The curve of the electric field energy density in the PE brush layer at the bottom of the nanoparticle with the concentration of the background salt solution at different distances between the particle and the plate at pH = 7.

**Figure 11:**
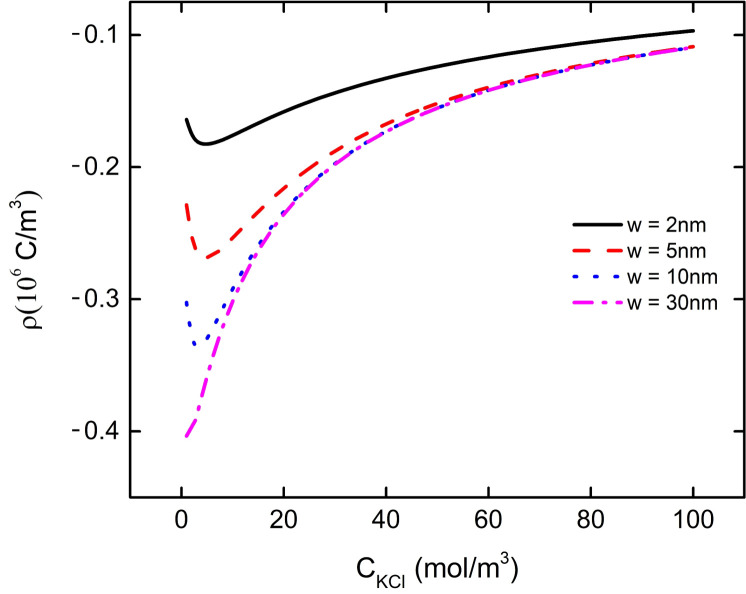
The curve of the total charge density in the PE brush layer at the bottom of the nanoparticle with the concentration of the background salt solution at different distances between the particle and the plate at pH = 7.

We found that the change of pH or the concentration of background salt solution will affect the electric field energy density in the PE brush layer at the bottom of the particle. When w = 2 nm, 5 nm, 10 nm, the electric field energy density at the bottom of the particle increases with the increase of the background salt solution concentration, which reaches the maximum value at the C_KCl_ = 10 mM, 7 mM, 3 mM, and then decreases with the increase of the background salt solution concentration, as shown in Figure 10. It can also be found from Figure 10 that the electric field energy density curve of w = 30 nm and w = ∞ basically overlaps (purple dotted line and green dot line in the Figure), which shows that when the distance between nanoparticle and the plate is more than 30 nm, there is no interaction between them. The same result can be found by observing the previous Figure 3 and Figure 6. We plot the curve of the total charge density in the PE brush layer at the bottom of the nanoparticles with the background salt solution under different particle and plate spacing to explain the change of the electric field energy density, as shown in Figure 11. From Figure 11, it can be seen that the curve of the total charge density in the PE brush layer at the bottom of the nanoparticle decreases with the increase of the background salt solution concentration, reaching the minimum value when the C_KCl_ is 10 mM, 7 mM and 3 mM respectively, and then increases continuously with the increase of the background salt solution concentration. This result corresponds to the result of electric field energy density in Figure 10. Due to the increase of the concentration of background salt solution, the concentration of K^+^ in the solution increases. Under the interaction between the particle and the plate, the local concentration of K^+^ at the bottom of the particle increases, leading to the decrease of the local concentration of H^+^, which increases the P–COO^−^ group in the PE brush layer and the bulk charge density of the PE brush layer. These phenomena eventually lead to the decrease of the total charge density curve in the PE brush layer at the bottom of the particle. When the curve of the total charge density decreases to the lowest point, the local concentration of K^+^ at the bottom of the particles increases with the increase of the background salt solution concentration, which weakens the influence of the bulk charge density of the PE brush layer (obtained from Equation (1)), resulting in the total charge density curve rising with the increase of the background salt solution concentration. These phenomena can be used to explain that the transport efficiency of nanoparticles in nanopores or nanochannels varies with the concentration of background salt solution.

## 4. Conclusions

In this paper, the effect of charge/discharge in the nanoparticle brush layer under different distance and background salt environments was investigated in terms of the interaction between PE brush layer modified nanoparticle and plate. The changes of charge density and electric field energy density of the PE brush layer at the bottom of the nanoparticles’ different distances between particle and plate were studied theoretically by numerical calculation from pH and background salt solution concentration. The results show that, (1) when the distance between the particle and the plate is close, the local H^+^ dissipation or enrichment at the bottom of the particle is large under the intense interaction, which results in the bulk charge density of the brush layer at the bottom of the particle being less than the top of the particle. For example, when w = 2 nm, the ratio between the bottom and the top of the brush bulk charge density of the particles is about 0.69. Under intense interaction, the electric field energy density in the brush layer at the bottom of the particle is low. With the increase of the distance between the particle and the plate, the charge effect will be produced on the bottom of the particle, which leads to the increase of the electric field energy density in the brush layer. When w ≥ 30nm, there is no interaction between the particle and the plate. At the same time, the electric field energy density at the bottom of particles increases first and then decreases with the increase of pH. (2) Under the fixed pH = 7, the PE brush layer is negatively charged, and the local cation at the bottom of the particles are enriched by the interaction between the nanoparticle and the plate. When the concentration of the background salt solution is small, the total charge density in the brush layer at the bottom of the particle is mainly affected by the local concentration of H^+^. With the increase of the concentration of background salt solution, the local K^+^ concentration at the bottom of the particle increases, which weakens the effect of local H^+^ concentration on the total charge density. Finally, the electric field energy density in the brush layer at the bottom of the particle increases first and then decreases with the increase of the background salt concentration. The above conclusions can provide theoretical support for the movement mechanism of macromolecular organic compounds in the nanochannel and the charge/discharge when they interact with the channel wall.
